# Anti-Amyloid Immunotherapies for Alzheimer’s Disease: A 2023 Clinical Update

**DOI:** 10.1007/s13311-023-01405-0

**Published:** 2023-07-25

**Authors:** Golnaz Yadollahikhales, Julio C. Rojas

**Affiliations:** Memory and Aging Center, Department of Neurology, Weill Institute for Neurosciences, University of California, 1551 4th Street, 411G, San Francisco, CA 94158 USA

**Keywords:** Amyloid, Alzheimer’s disease, Clinical trial, Disease-modifying therapy, Preclinical

## Abstract

The amyloid cascade hypothesis is a useful framework for therapeutic development in Alzheimer’s disease (AD). Amyloid b_1-42_ (Aβ) has been the main target of experimental therapies, based on evidence of the neurotoxic effects of Aβ, and of the potential adverse effects of brain Aβ burden detected in humans in vivo by positron emission tomography (PET). Progress on passive anti-amyloid immunotherapy research includes identification of antibodies that facilitate microglial activation, catalytical disaggregation, and increased flow of Aβ from cerebrospinal fluid (CSF) to plasma, thus decreasing the neurotoxic effects of Aβ. Recently completed phase 2 and 3 trials of 3rd generation anti-amyloid immunotherapies are supportive of their clinical efficacy in reducing brain Aβ burden and preventing cognitive decline. Data from recent trials implicate these agents as the first effective disease-modifying therapies against AD and has led to the US Food and Drug Administration (FDA) recent approval of aducanumab and lecanemab, under an accelerated approval pathway. The clinical effects of these agents are modest, however, and associated with amyloid-related imaging abnormalities (ARIA). Testing the effects of anti-Aβ immunotherapies in pre-symptomatic populations and identification of more potent and safer agents is the scope of ongoing and future research. Innovations in clinical trial design will be the key for the efficient and equitable development of novel anti-Aβ immunotherapies. The progress in the field of AD therapeutics will bring new clinical, logistical, and ethical challenges, which pose to revolutionize the practice of neurology, dementia care, and preventive cognitive healthcare.

## The Amyloid Cascade Hypothesis as a Framework for Therapeutic Development in Alzheimer’s Disease

**Fig. 1:**
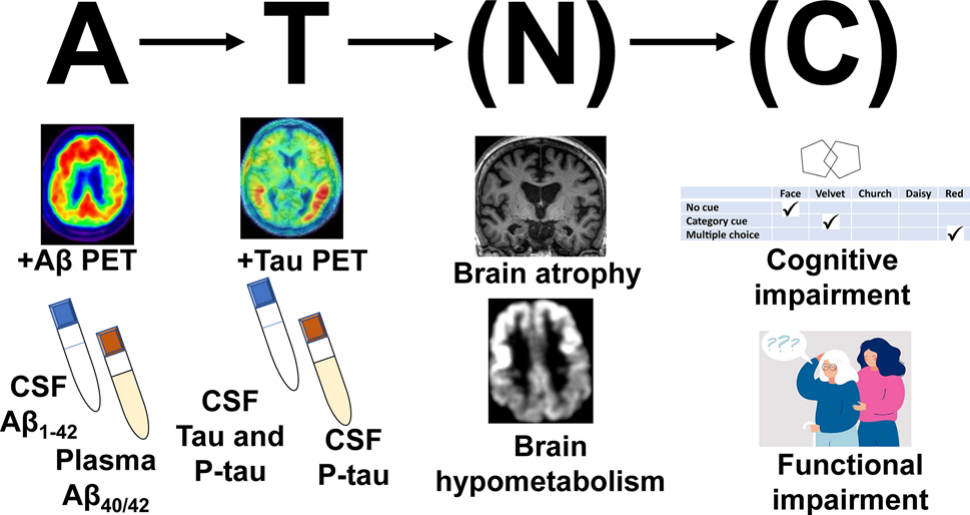
The amyloid cascade hypothesis and available means to determine a patient’s status. The hypothesis proposes a central role of Aβ in the pathophysiology of Alzheimer’s disease. Amyloid deposition (A) leads to tau deposition (T), which in turn is the substrate for selective neurodegeneration (N) and cognitive impairment (C). Neurodegeneration and cognitive impairment appear in parentheses to highlight that they may or not occur in the presence of Aβ and tau pathology. Alzheimer’s disease is thus, a pathobiological concept, and may be targeted before neurodegeneration or cognitive symptoms occur. See reference 12 by Jack et al. [12]

The predominant mechanistic model of Alzheimer’s disease (AD) has emerged from the key observation that amyloid plaques are a constant neuropathological feature of the disease, as originally described by Alzheimer in early-onset cases [1] and by Fischer in late-onset cases [2]. Alzheimer and Fischer were careful not to attribute amyloid plaques a definitive causative role in the neurodegenerative process underlying the dementia cases they described. A notion that amyloid plaques cause AD emerged in the 1960s, after the rediscovery of their work, modern neuropathological studies at the time, and the recognition that cases of early and late-onset dementia both feature amyloid plaques neuropathology [3, 4]. This notion positioned amyloid plaques as a logical target for experimental therapies in AD. Although amyloid plaques may or may not be viewed as the ultimate cause of AD, preclinical studies and longitudinal clinical investigations have provided undisputable evidence that amyloid β_1-42_ (Aβ) accumulation is a key early pathophysiological event in AD, in great part supported by the introduction of brain amyloid imaging with positron emission tomography (PET) [5–8]. The “amyloid cascade hypothesis” poses that Aβ accumulation is a central early pathogenic event in AD and leads to neurodegeneration and cognitive impairment through its induction of aberrant accumulation of the microtubule-associated protein tau [9, 10]. According to its original conception, a high rate of Aβ accumulation disrupts calcium homeostasis and induces tau hyperphosphorylation, which is highly neurotoxic and forms paired helical filaments that accumulate in the form of intraneuronal neurofibrillary tangles. In 2018, an NIH/NIA-commissioned working group led by experts in the field of AD experimental therapeutics introduced a contemporary view of the “amyloid cascade hypothesis,” in which Aβ accumulation occurs slowly over years, which helps to explain the presence of Aβ without degeneration or cognitive impairment, as surprisingly found in early PET amyloid imaging studies in humans [11, 12]. This allows understanding AD as a sequential pathobiological process, not equivalent to a clinical syndrome or a neuropathological state [12]. Thus, this contemporary view of the “amyloid cascade hypothesis” constitutes a pragmatic theoretical framework to maximize the chances of successfully identifying effective disease-modifying therapies, amenable to use even in prodromal or pre-symptomatic stages (Fig. 1). This view aligns with current available data, but alternate hypotheses of neurodegeneration in AD, in which amyloid an/or tau do not play a significant pathobiological role are possible [12].

The most compelling evidence justifying the use of the “amyloid cascade hypothesis” as the mainstream theoretical framework to guide therapeutic development in AD include the following: (1) the in vitro and in vivo evidence of the neurotoxic effects of diverse amyloid species, especially Aβ and (2) the clinical evidence of high risk of cognitive decline in individuals with brain Aβ burden.

### Neurotoxic Effects of Amyloid Species

The Aβ peptide is generated by metabolism of amyloid precursor protein (APP). Normally, APP is cleaved close to the cell membrane by α-secretase, with production of a soluble extracellular fragment, sAPPα. Alternatively, APP may be cleaved by β-secretase (or β site APP cleaving enzyme 1, BACE1) generating a soluble extracellular fragment (sAPPβ) and a cell-membrane-bound fragment (C99). C99 is cleaved within the membrane by γ-secretase, an enzymatic complex formed by presenilin (PSEN), nicastrin, anterior pharynx-defective 1, and presenilin enhancer 2. Presenilin is the catalytic subunit of γ-secretase and is encoded by either the *PSEN1* or *PSEN2* genes. The γ-secretase cleavage releases an intracellular peptide known as amyloid intracellular domain (AICD) and the Aβ peptide. Aβ may have different lengths, the most abundant being of 40 amino acids (Aβ_1–40_) and the less soluble of 42 amino acids (Aβ_1–42_). Aβ monomers aggregate to form oligomers, protofibrils, fibrils, and ultimately plaques that represent one of the hallmarks of AD pathology [13].

Although amyloid plaques were initially believed to be the only pathogenic form of Aβ, studies have shown that Aβ oligomers (AβOs) injected intraventricularly to naive animals have negative effects on memory function [14]. AβOs instigate tau pathology [15], loss of neuronal polarity [16], impairment of axonal transport [17], deterioration of synapses [18], oxidative stress [19], endoplasmic reticulum stress [20], insulin resistance [21], neuroinflammation [22], cholinergic impairment [23], loss of trophic factors [24], epigenetic changes [25], ectopic mitosis [26], and selective nerve cell death [27]. Also, AβOs as gain-of-function pathogenic ligands can bind adventitiously to specific proteins acting as toxin receptors [28].

In addition to AβOs, a myriad of truncated Aβ peptides have also been found to likely play a role in AD pathogenesis. Truncated Aβ peptides can form stable oligomeric complexes with the full-length Aβ peptide [29]. In fact, N-terminally truncated Aβ peptides formed through pyroglutamylation of glutamic acid residues are increasingly recognized as very toxic species of Aβ. Aβ pyroglutamylation increases the aggregation speed of Aβ and drives misfolding of Aβ into more toxic aggregates [30, 31]. Similarly, Aβ protofibrils are highly toxic species that generate reactive oxygen species, chronic neuroinflammatory responses, and impairment of synaptic function [32].

### Consequences of Aβ Burden in Humans

The amyloid hypothesis has gained supportive evidence with the development of amyloid PET, which has allowed detection and quantification of brain Aβ accumulation in vivo. Early studies documented high brain Aβ load in amnestic dementia, compared to individuals with mild cognitive impairment (MCI) and cognitively healthy individuals. This neuroimaging technique showed the potential to discern living individuals with dementia and underlying brain Aβ accumulation, from individuals with dementia without Aβ accumulation, who are presumed to have dementia caused by etiologies other than AD. The relevance of this finding for clinical trials is that patients can be selected to participate in studies based on their brain amyloid status, so prospective disease-modifying therapies could be offered to individuals actually affected by AD.

**Fig. 2:**
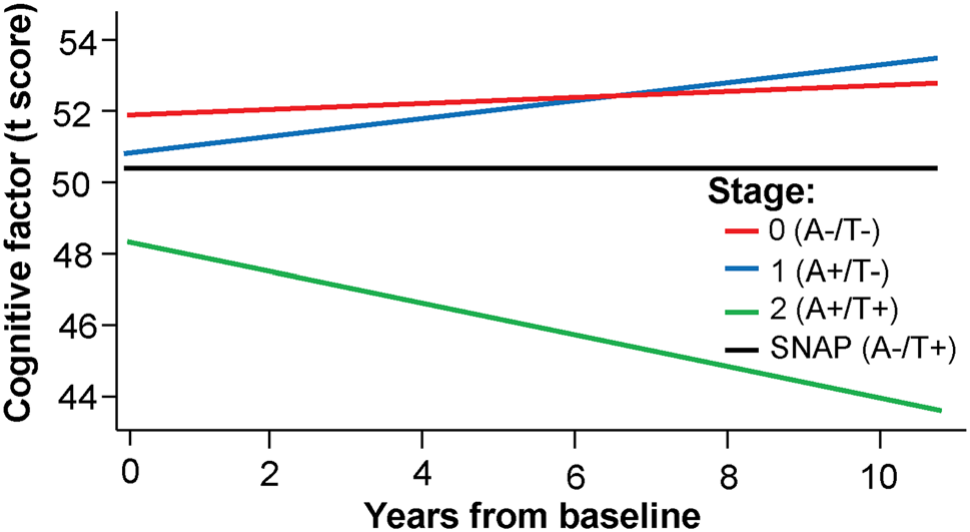
Estimates of longitudinal change in cognition among individuals classified into the 3 preclinical AD groups (stages 0, 1, 2) and SNAP (As in reference 33). Only individuals with cognitively healthy Alzheimer’s disease defined as the presence of positive Aβ *and* tau biomarkers showed cognitive decline. Abbreviations: SNAP suspected non–Alzheimer disease, A Amyloid, T Tau

Another important observation from these early amyloid neuroimaging studies was that about 30% of cognitively healthy individuals had a positive amyloid PET. This supports that AD pathophysiology appears years before cognitive impairment, and disease-modifying therapies could have a preventive role if offered in pre-symptomatic stages to individuals with evidence of high brain Aβ burden [11]. A meta-analysis of four cohorts that combined 800 cognitively healthy participants showed that not only abnormal cerebrospinal fluid (CSF) Aβ but also CSF abnormalities in tau or P-tau181 are important predictors of cognitive decline. Only those cognitively healthy individuals with abnormalities in Aβ (A +) and tau or P-tau181 (T +) showed slow cognitive decline over the next 10 years, whereas cognition in individuals without combined amyloid and tau abnormalities (A − /T − , A + /T − or A − /T +) maintained stable cognition over the same time (Fig. 2) [33]. Finally, longitudinal amyloid PET data from older cognitively healthy adults have revealed that it is the increased rate of Aβ accumulation that subsequently increases the rate of tau accumulation (the so-called “caTAUstrophe”), which in turn determines the cognitive change. This sequence of events explains about 45% of the cognitive status change at 7 years and is more determinant than the baseline Aβ or tau brain load [34]. These studies suggest that Aβ may play an important early role in AD pathogenesis and may be an important therapeutic target, although they also highlight the possibility that other factors are at play as well, which may help to explain the somewhat modest effect of anti-Aβ therapies.

## Immunotherapy as a Therapeutic Strategy in the Context of the Amyloid Cascade Hypothesis

The therapeutic approaches available for AD therapeutic development are determined in great part by the available pharmacological technology. The traditional approaches rely on small molecules that act as receptor agonist/antagonists, enzymatic inhibitors, or cofactors, and the future may bring clinical genetic manipulation via anti-sense oligonucleotides, gene therapy and gene editing, or non-invasive bioenergetic manipulations. Nevertheless, the most rapidly evolving therapeutic mechanism of our times is immunotherapy. In particular, antibody-based immunotherapy is not only used in AD experimental therapeutics targeting Aβ but is also implemented as a successful strategy in other fields of medicine, such as neuroimmunology, rheumatology, and oncology. Broadly conceptualized, immunotherapy consists of the manipulation of the humoral or cellular components of the immune system to induce a beneficial effect. Antibody-based immunotherapy can be active or passive.

### Active Anti-Amyloid Immunotherapy

Active immunization uses an immunogen, in this case Aβ, combined with an adjuvant to stimulate an immune response. The subject’s immune system generates anti-Aβ antibodies that mediate Aβ clearance or prevention of its deposition. Active immunization against Aβ was adapted as a strategy in human AD clinical trials, based on transgenic mouse model studies that supported that active immunization reduces Aβ deposition and cognitive decline [35]. AN1792 was a human trial that involved up to 5 immunizations over a 36-week period. It was suspended due to occurrence of subacute meningoencephalitis (inflammation of the brain and meninges) in approximately 6% of patients. This side effect was attributed to unregulated activation of T and B lymphocytes [36]. In order to overcome the adverse effects, subsequent trials tested alternate adjuvants or immunization protocols such as intranasal or subcutaneous administration [37]. For example, a follow-up phase 2a study of AN1792 identified that, at 4.6 years, responders maintained low but detectable anti-AN1792 antibody titers and had significantly reduced functional decline compared to placebo-treated patients. Brain volume loss in antibody responders was not significantly different from placebo-treated patients, and no further cases of encephalitis were detected [38]. Active anti-amyloid immunotherapy trials, including novel DNA-based vaccine technologies, are currently studied in at least three phase 1 studies and two phase 2 studies in MCI or mild dementia [39–43]. Prevention trials with active anti-amyloid immunotherapy in middle-age cognitively healthy individuals before brain amyloid accumulation may also be possible in the near future.

### Passive Anti-Amyloid Immunotherapy

Passive immunotherapy involves the in vitro production of monoclonal anti-Aβ antibodies and then direct infusion of these antibodies into subjects [44]. This approach allows greater control of dose and a mechanism to withdraw treatment should any adverse events become apparent. Several downstream mechanisms have been proposed to mediate the beneficial effects of passive anti-amyloid immunotherapy. Microglial activation and removal of amyloid plaques have been observed after direct injection of anti-Aβ antibodies into the brains of Tg2576 mice [45]. Catalytic disaggregation has been suggested by disruption of the tertiary structure of the plaque and subsequent disaggregation that result from the interaction between an antibody and an Aβ deposit [35]. The peripheral sink mechanism is proposed based on studies using anti-Aβ antibodies which were specifically designed to not bind to Aβ plaques in the brain (i.e., the m266 antibody). When these antibodies were administered by intraperitoneal injection in the PDAPP mouse, a rapid 1000-fold increase in circulating plasma Aβ levels were observed. These data suggested that circulating Aβ antibodies bind to plasma Aβ and transiently reduce the circulating levels of soluble Aβ. In turn, this reduction promotes the removal of soluble Aβ from the brain by mass action transfer across the blood–brain barrier to the vasculature, hence the term peripheral sink [46]. Improved behavioral performance and electrophysiological benefits potentially mediated by the peripheral sink have been documented in aged PDAPP mice, with increases of plasma Aβ, and only partial decreases in brain amyloid plaque [47].

The development of passive anti-amyloid immunotherapies has included extensive preclinical work to target epitopes in the N-terminus, C-terminus, or mid region of the Aβ protein and to make variations in the IgG isotypes to modulate immunogenicity [48]. For example, N-terminal antibodies administered to aged APP23 mice caused a significant increase in the occurrence of cerebral amyloid angiopathy-associated microhemorrhage as well as acute hematomas [49]. In contrast, m266, an IgG1 antibody against the Aβ mid-domain, reduced brain Aβ deposits in PDAPP mice without causing microhemorrhages [50].

## Initial Human Clinical Trials of Anti-Amyloid Immunotherapies

**Fig. 3:**
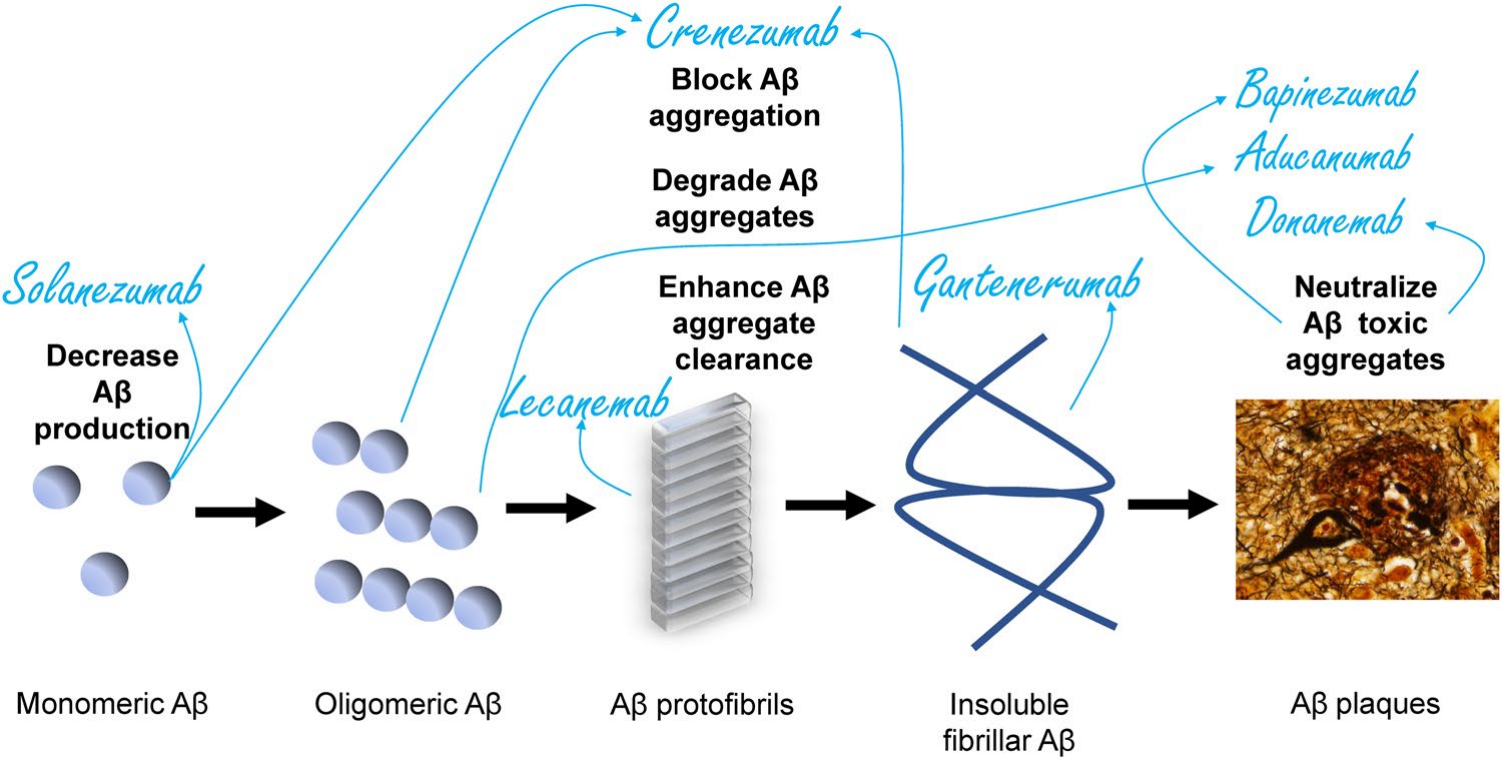
Mechanisms of action of different anti-amyloid antibodies in relation to stages of Aβ aggregation

The extensive preclinical work on amyloid biology allowed the expansion of the anti-amyloid immunotherapy armamentarium to target different Aβ species: monomers, oligomers, protofibrils, insoluble fibrils, and insoluble plaques. A first-generation approach (bapineuzumab) targeted the N-terminus of the aggregated Aβ. Crenezumab, gantenerumab, and solanezumab are second generation agents that were developed to target non-plaque Aβ species (Table 1). Third generation agents were developed as high affinity antibodies against Aβ protofibrils (lecanemab) or plaque Aβ (aducanumab and donanemab) (Table 2 and Fig. 3).

**Table 1:** Negative phase 3 anti-amyloid immunotherapy trials in symptomatic AD

	***Aducanumab**** (ENGAGE)*		***Solanezumab*** (*EXPEDITION 2*)		***Gantenerumab*** (*GRADUATE I*)		**Crenezumab** (CREAD 2)	
	Mean difference vs. placebo (95% CI)	*P* value	Mean difference vs. placebo (95% CI)	P value	Mean difference vs. placebo (95% CI)	*P* value	Mean difference vs. placebo (95% CI)	*P* value
CDRsb	0.03 (− 0.26 to 0.33)^a^	0.833	− 0.3 (− 0.7 to 0.2)	0.17	0.31	0.095	1.30 (− 0.00 to 2.6)^a^	0.05
MMSE	− 0.1 (− 0.6 to 0.5)	0.811	0.8 (0.2 to 1.4)	0.01	0.32	0.290	− 0.41 (− 2.42 to 1.60)	
ADAS-Cog	− 0.59 (− 1.61 to 0.43)^b^	0.258	− 1.3 (− 2.5 to 0.3)^a,c^	0.06	1.25	0.054	1.74 (− 2.40 to 5.89)	
ADCS-ADL	0.7 (− 0.2 to 1.6)	0.15	1.6 (− 0.2 to 3.3)^a^	0.08	1.11	0.133	− 2.52 (− 8.74 to 3.70)	
NPI	0.1	0.907	− 0.2 (− 1.8 to 1.5)	0.85	− .086		− 0.76 (− 1.75 to 0.23)	
PET amyloid (composite SUVR)	− 0.232 (− 0.256 to − 0.208)	< 0.0001	N/A		− 57.4 (centiloids)	< 0.0001	0.021 (− 0.030 to 0.037)	0.39
Total Aβ42 in CSF	Increased	< 0.001	402.8 (1628.4 to 4437.9)	< 0.001	34 (% change from baseline)	< 0.0001	− 308.2 (− 367.4 to –249.1)	< 0.001

*CDRsb* clinical dementia rating-sum of boxes, *MMSE* mini-mental state examination, *ADAS-Cog* Alzheimer’s disease assessment scale-cognitive, *ADCS-ADL* Alzheimer’s disease cooperative study activities of daily living inventory, *ADCS-iADL* Alzheimer’s disease cooperative study-instrumental activities of daily living inventory, *PET* positron emission tomography, *SUVR* standardized uptake value ratio, *CI* confidence interval

^a^Primary outcome for this trial

^b^ADAS-Cog13 was used in ENGAGE, GRADUATE I, and CREAD

^c^ADAS-Cog11 was used in EXPEDITION

**Table 2:** Clinical effect of 3rd generation anti-amyloid immunotherapies

	***Aducanumab*** (EMERGE trial)		***Donanemab*** (TRAILBLAZER-ALZ)			***Lecanemab*** (clarity AD)			
	Most effective dose difference vs. placebo	Placebo change ± SE	Most effective dose difference vs. placebo		Placebo change ± SE	Most effective dose difference vs. placebo	Placebo change ± SE		
Primary outcome									
CDRsb	− 0.39 (− 22%)	1.74 ± 0.11	iADRS	3.20 (25%)	− 10.06	CDRsb	− 0.45 (− 27%)	1.66	
95% CI	− 0.69, 0.09		95% CI	0.12, 6.27		95% CI	− 0.67, 0.23		
*P* value	0.012		*P* value	0.04		*P* value	< 0.001		
Secondary outcomes									
MMSE	0.6 (− 18%)	− 3.3 ± 0.2	MMSE	0.64		ADCOMS	− 0.05	0.214	
95% CI	0.0, 1.1		95% CI	− 0.40, 1.67		95% CI	− 0.074, − 0.027		
*P* value	0.049		*P* value			*P* value	< 0.001		
ADAS-Cog13	− 1.40 (− 27%)	5.16 ± 0.40	ADAS-Cog13	− 1.86		ADAS-Cog14	− 1.44	5.58	
95% CI	− 2.46, − 0.34		95% CI	− 3.63, − 0.09		95% CI	− 2.27, − 0.61		
*P* value	0.010		*P* value			*P* value	< 0.001		
ADCS-ADL-MCI	1.7 (− 40%)	− 4.3 ± 0.4	ADCS-iADL	1.21		ADCS MCI-ADL	2	− 5.5	
95% CI	0.7, 2.7		95% CI	− 0.77, 3.20		95% CI	1.2, 2.8		
*P* value	< 0.001		*P* value			*P* value	< 0.001		
NPI	− 1.3 (− 87%)		CDRsb	− 0.36					
95% CI			95% CI	− 0.83, 0.12					
*P* value	0.022		*P* value						
PET amyloid (composite SUVR)	− .278 (− 71%)	4% increase	PET amyloid (centiloids)	− 85.06	0.93	PET amyloid (centiloids)	− 59.1	3.64	
95% CI	− 0.306, − 0.250		95% CI	− 92.6, − 77.4		95% CI	− 62.2, − 55.6		
*P* value	< 0.0001		*P* value			*P* value	< 0.01		

*CDRsb* clinical dementia rating-sum of boxes, *MMSE* mini-mental state examination, *ADAS-Cog13* Alzheimer’s disease assessment scale-cognitive subscale-13 items item, *ADCS-ADL-MCI* Alzheimer’s disease cooperative study activities of daily living inventory-mild cognitive impairment, *ADCS-iADL* Alzheimer’s disease cooperative study-instrumental activities of daily living inventory, *ADCOMS* Alzheimer’s disease composite score, *iADRS* integrated Alzheimer’s disease rating scale, *SE* standard error, *PET* positron emission tomography, *SUVR* standardized uptake value ratio, *CI* confidence interval

### First Generation (N-Terminus Aggregated Plaque)

*Bapineuzumab* is a humanized murine antibody against residues 1–5 at the N-terminus of the Aβ protein [51]. This unique specificity of bapineuzumab, an anti-3D6 humanized antibody, precludes recognition of unprocessed APP. Furthermore, the 3D6 epitope is detectable in all forms of Aβ, from compacted β-amyloid plaques in AD to soluble oligomeric species [52]. This antibody induces Fc-receptor-mediated microglial phagocytosis, with neutralization and removal of Aβ plaques. In a phase 3 study with 1331 individuals with mild or moderate AD, with a mini-mental state examination (MMSE) score of 16 to 26, and a Hachinski Ischemic scale score ≤ 4, bapineuzumab showed no difference in the primary cognitive outcome, compared to placebo, but it induced reductions in Aβ accumulation [51]. This negative result may be related to low potency for plaque removal with bapinezumab or disease progression despite plaque removal, which was not determined. Moreover, given that brain amyloid imaging was not a widely available imaging technique at the time, brain PET amyloid status was not used to enroll participants in this early study. This may have contributed to enrollment of participants with dementia not associated with brain amyloid accumulation, possibly confounding the results and preventing determination of the effectiveness of bapinezumab as an anti-amyloid therapy. Recent studies support, indeed, that about 50% of participants with MCI and about 30% of those with dementia diagnosed on clinical grounds only are amyloid brain PET negative [53, 54].

### Second Generation (Non-Plaque Aβ Forms)

*Solanezumab* recognizes a linear epitope of the mid-domain (residues 16–26) Aβ molecule, which is buried in oligomers and fibrils, so it targets only soluble monomeric Aβ [55, 56]. Solanezumab facilitates the “peripheral sink,” with flow of Aβ from the brain to plasma and decreases synaptic toxicity. In phase 3 studies of mild and moderate AD (EXPEDITION and EXPEDITION 2), solanezumab was well-tolerated, with essentially no incidence of amyloid-related imaging abnormalities (ARIA) attributable to the drug, although the agent did not affect the decline in cognition or function compared to placebo [57]. Solanezumab was tested in A4, a phase 3 trial of pre-symptomatic AD that recruited participants based on a positive brain amyloid PET. This was a negative trial, since solanezumab did not slow cognitive decline on the primary outcome measure, the preclinical Alzheimer cognitive composite (PACC). The PACC was developed to measure the aspects of cognitive decline relevant in preclinical AD, and it is an equally weighted composite that tests episodic memory, timed executive function, and global cognition. Brain amyloid measured by PET continued to accumulate over time in both the placebo and solanezumab groups. The adverse ARIA-E events were also similar in between both groups [58].

*Gantenerumab* targets insoluble fibrillar Aβ and recognizes a conformation-specific N-terminal to mid-domain region of the protein including residues 3–11 and 18–27, with sub-nanomolar affinity [59]. Gantenerumab reduces fibrillar Aβ by activating glia and inducing phagocytosis. In a phase 1 study, subcutaneous gantenerumab induced ARIA in two apolipoprotein (*APO)E* e4 carriers, with partial Aβ reduction in the same brain regions where ARIA developed [60]. Phase 2 and 3 studies in patients with mild AD were stopped due to futility, as no clinical benefit was observed, despite an increase in CSF Aβ and a numeric decrease in total tau. Also, ARIA incidence increased in a dose- and *APOE* ε4 allele-dependent manner [61]. Recently, two phase 3 trials, GRADUATE I and II, of subcutaneous gantenerumab in participants with MCI and mild AD treated for 27 months did not reach the primary end point of slowing clinical decline on CDRsb at week 116 [62]. Moreover, the secondary clinical outcome measures of cognition (ADAS-Cog13 and MMSE) and function (ADCS-ADL and FAQ) also did not show any significant favorable change. Only 28% of participants in GRADUATE I and 27% in GRADUATE II showed a reduction in amyloid levels below the positivity threshold at week 116. Tau PET showed continuous tau accumulation in both arms and no treatment effect, although the sample size was small. Volumetric MRI showed increased reduction in brain volume, whereas hippocampal volume was not affected after gantenerumab treatment. Robust treatment effects were observed in fluid biomarkers including both plasma (pTau181, pTau217, Aβ40, and Aβ42) and CSF. Symptomatic ARIA-E was seen in 5% of the participants on gantenerumab, whereas ARIA-H happened in 22.9% of cases [61, 63, 64].

*Crenezumab* is a fully humanized monoclonal antibody that binds to multiple Aβ forms, monomeric, oligomeric, and fibrillar, although it has a tenfold higher affinity for oligomers over monomers [65]. It was designed with an IgG4 class backbone to reduce inflammatory cytokine release from microglia and complement activation, while preserving phagocytosis. A phase 2 study showed that early disease benefited the most from crenezumab, with a reduction of cognitive decline of 35% in MCI vs. 23.8% in mild AD vs. 16.8% in mild-to-moderate AD. This study also showed that intravenous crenezumab was superior to subcutaneous administration [66]. Crenezumab did not reduce cognitive decline in two phase 3 studies of mild AD [67]. Evaluation of crenezumab in cognitively unimpaired members of the Colombian presenilin 1 (PSEN1) E280A kindred in Alzheimer’s prevention initiative autosomal-dominant Alzheimer’s disease (API ADAD) trial was negative on primary and secondary end points, and further development was discontinued [68].

## Third-Generation Anti-Amyloid Immunotherapies in Active Clinical Dsevelopment

### Aducanumab

Aducanumab (BIIB037) is an anti-Aβ immunotherapy that targets residues 3–7 in the Aβ N-terminus [69]. Although this epitope is exposed in monomers, oligomers, and fibrils, the antibody is highly selective for oligomeric or fibrillar aggregates based on low monovalent affinity and strong avidity for epitope-rich aggregates [70]. Preclinical studies showed that intraperitoneal aducanumab (single dose, 30 mg/kg) binds to diffuse and compact Aβ plaques in the brains of 22-month-old female Tg2576 transgenic mice. Aducanumab did not affect plasma or brain Aβ concentrations consistent with the observation that aducanumab does not bind to soluble Aβ monomers. Aducanumab reduced all forms and sizes of Aβ deposits by up to 70%, including dose-dependent reductions in the cortex and hippocampus. The mechanism of Aβ clearance is binding of microglia to the Fc antibody region, causing enhanced phagocytosis of aducanumab–Aβ complexes [71–73]. Aducanumab also limits oligomers neurotoxicity by blocking the binding of soluble Aβ oligomers to metabotropic receptors and by slowing their release into the neuropil from plaques [74] preventing calcium-induced neurotoxicity [75, 76].

Four phase I trials were done to evaluate the pharmacokinetics, safety, or tolerability of aducanumab in MCI or mild dementia due to AD. The first phase I randomized, double-blind, placebo-controlled, single ascending-dose multicenter study enrolled a total of 53 patients with mild-to-moderate AD, who were sequentially randomized 6:2 to aducanumab (0.3, 1, 3, 10, 20, 30, and 60 mg/kg) or placebo. Doses ≤ 30 mg/kg were tolerated with no severe or serious adverse events. All three patients who received 60-mg/kg aducanumab developed symptomatic amyloid-related imaging abnormalities, which completely resolved by weeks 8–15 [77]. The other relevant phase I study was PRIME (*n* = 165), which was a phase 1b study with participants enrolled based on a positive amyloid PET and randomized to placebo or monthly intravenous aducanumab at 1, 3, 6, or 10 mg/kg for 1 year. Aducanumab at 3, 6, and 10 mg/kg reduced brain Aβ plaques amyloid PET at 1 year. The 10 mg/kg group had brain Aβ clearance almost compatible with a negative amyloid PET (mean pos-treatment standardized uptake value ratio (SUVR) composite score of 1.16 (normal ≤ 1.10)) [78]. On exploratory clinical outcomes at 1 year, aducanumab induced significant dose-dependent slowing of clinical progression, as measured by the change in the clinical dementia rating scale-sum of boxes (CDRsb) scores, which were adjusted for baseline CDRsb and *APOE* e4 status, with the greatest slowing observed in the 10-mg/kg dose arm. The study was limited by a staggered parallel-group design, small sample sizes, recruitment in a single geographical region (US only), and possible unblinding due to incidence of ARIA [73].

Aducanumab was subsequently tested in EMERGE (*n* = 1638) and ENGAGE (*n* = 1647), two identically multicenter, randomized, double-blind, placebo-controlled phase 3 trials in MCI and mild dementia [79]. Participants were recruited based on a positive amyloid PET by visual inspection, and they were randomized (1:1:1) to intravenous aducanumab 6 mg/kg, 10 mg/kg, or placebo every 4 weeks for 76 weeks. For the first time, *APOE* genotype was used to stratify titration regimens as a consideration for the risk of ARIA, with participants in both dose regimens receiving lower doses than the target dose for the first 3–6 infusions if they were *APOE* ε4 carriers. The studies were also designed to have dosing affected by detection of ARIA as the studies progressed. Continuation, temporary suspension or permanent discontinuation occurred based on the type, size, course, and clinical correlates of ARIA.

The primary clinical outcome was met in EMERGE. At 76 weeks, aducanumab 10 mg/kg induced a difference of − 0.39 (95% CI − 0.69 to − 0.09, *p* = 0.012) in disease severity, measured with the CDRsb, compared to placebo, which represented a 22% reduction in clinical progression. Aducanumab 10 mg/kg also induced less decline vs. placebo on secondary endpoints including MMSE (0.6, 95% CI 0.0 to 1.1, *p* = 0.049, or 18% reduction in decline), Alzheimer’s disease assessment scale-cognitive subscale-13 items (ADAS-Cog13, − 1.40, 95% CI − 2.46 to − 0.34, *p* = 0.010, or 27% reduction in decline), the Alzheimer’s disease cooperative study activities of daily living inventory-mild cognitive impairment scale (ADCS-ADL-MCI, 1.7, 95% CI 0.7 to 2.7, *p* < 0.001, or 40% reduction in decline), and less worsening in the neuropsychiatric inventory (NPI-10, − 1.3, *p* = 0.022 or 87% reduction of worsening) (Table 1).

In contrast, clinical outcomes were not met in ENGAGE, with no differences between treatment groups and placebo in primary or secondary outcomes. Nevertheless, in both EMERGE and ENGAGE, there was a dose–response effect on brain amyloid PET SUVR at 78 weeks. The mean decrease in brain amyloid compared to baseline was 71% in EMERGE and 59% in ENGAGE, whereas the change in brain amyloid in the placebo group was 4% increase and 1% decrease, in each study respectively. At 78 weeks, 48% of patients from EMERGE and 31% of patients from ENGAGE treated with aducanumab 10 mg/kg had a PET composite SUVR score compatible with a negative brain amyloid PET (SUVR ≤ 1.10). Also, in both EMERGE and ENGAGE, aducanumab 10 mg/kg reduced plasma P-tau181 by 13% and 16%, respectively. In contrast, placebo groups showed increases in plasma P-tau181 of 8% and 9%, respectively.

Based on combined data from both EMERGE and ENGAGE, ARIA, both with edema (ARIA-E) and/or hemorrhage (ARIA-H) combined was common (41.3% with aducanumab 10 mg/kg), but the incidence of symptomatic ARIA was low, with only about of 20% of radiographic ARIA cases showing mild symptoms, most commonly headache. The original report and a subsequent combined safety analysis [79, 80] describe incidences of symptomatic ARIA-E, but not ARIA-H, because symptomatic ARIA-H does not tend to occur in isolation (i.e., it commonly occurs in conjunction with ARIA-E). In our experience with aducanumab and the other anti-amyloid immunotherapies, isolated ARIA-H in the form of microhemorrhage or superficial siderosis, is usually asymptomatic. The incidence of ARIA-E was higher in the 10 mg/kg groups, compared to the 6 mg/kg groups (35% vs. 26%, in EMERGE, and 36% vs. 26% in ENGAGE), and in *APOE* ε4 carriers compared to noncarriers (43% vs. 18% in EMERGE, and 42% vs. 23% in ENGAGE) (Table 2). Of all cases with ARIA-E, a minority were symptomatic (20% in EMERGE and 29% in ENGAGE). Common symptoms included headache, confusion, dizziness, and nausea. The majority of new ARIA-E occurred during the first eight doses (69.1% in EMERGE and 77.4% in ENGAGE) [79]. ARIA-H with aducanumab 10 mg/kg occurred in a balanced way in both studies, in the form of microhemorrhage (19.1%), superficial siderosis (14.7%), or hemorrhage > 1 cm^3^ (0.3%) [80]. Serious adverse ARIA events (e.g., delirium, memory worsening, seizures) were uncommon regardless of *APOE* ε4 genotype (1.5% in EMERGE and 1.4% in ENGAGE). The separate incidence of symptomatic ARIA-H is not reported, but the incidence of symptomatic ARIA-E was similar to the incidence of ARIA-E or ARIA-H combined in both studies. No fatal events attributed to any form of ARIA were observed in either study [79, 80].

The difference in clinical outcomes between EMERGE and ENGAGE, with one study meeting its primary outcome and the other not, led to a heated debate about the clinical efficacy among clinical trialists, dementia experts, patient advocates, regulatory agencies, including the FDA and the Centers for Medicare & Medicaid Services (CMS), and the media. Positions in favor of its approval highlighted the unlikelihood that EMERGE results are a false positive, given the consistency of its primary and secondary clinical outcomes and biomarker outcomes; the significance of a 0.39 points difference in the CDRsb, which may represent a clinically meaningful slowness in decline, especially in the MCI stage; the results that are in line with those of the phase I trial PRIME; and the results of phase 2 trials of other 3rd generation anti-amyloid agents (i.e., donanemab and lecanemab). Opinions against the approval of aducanumab emphasized the lack of replication of EMERGE results, the high incidence of side effects in light of seemingly modest clinical benefits, and the high cost of the drug that was felt to have potential for significant financial burden on medical systems and accentuation of disparities in dementia care [81].

The EMERGE and ENGAGE researchers proposed that halting the study after a pre-specified futility analysis limited capturing meaningful data. They discussed that futility analyses are introduced to prevent exposure of participants to ineffective treatments, but in the case of EMERGE and ENGAGE, two assumptions necessary for valid interim analyses were violated: (1) that treatment effects are different in the two studies and (2) that treatment effects will not change over time. The differences in outcomes between the two studies are obvious, and both showed a larger magnitude of treatment effects in the final dataset. Protocol amendments changed the target dose for about two-thirds of participants in the high dose groups, to the effect that only 22% of ENGAGE participants reach the high dose, as opposed to 29% of EMERGE participants. By the time study amendments were introduced, ENGAGE had enrolled 200 more patients that EMERGE [79].

Aducanumab (Aduhelm™, Biogen) was approved for the treatment of MCI or mild dementia by the FDA on June 7, 2021, under accelerated approval based on reduction of Aβ plaques. Its continued approval is contingent upon verification of clinical benefit in confirmatory trials. This makes it the first approved disease-modifying therapy for AD and the first approved drug indicated for the management of AD in 18 years. Experts recommend its use in patients with positive AD biomarkers and, in general, in those with clinical characteristics similar to patients enrolled in EMERGE and ENGAGE [82]. Expert recommendations for use include careful consideration of *APOE* genotype, baseline cerebral amyloid angiopathy burden assessed by structural brain MRI susceptibility weighted imaging sequences, and oral anticoagulant use, all of which may increase the risk of ARIA. CMS did not approve financial coverage of aducanumab, unless patients receiving it are enrolled in CMS-approved studies, such as surveillance clinical trials or registries [83]. Until the time of this manuscript writing, this policy will apply to all new drugs in the anti-amyloid immunotherapy class that receive FDA approval. As part of this surveillance effort, EMBARK is a phase 3β study that evaluates the safety and tolerability of intravenous aducanumab 10 mg/kg every 4 weeks in patients who had previously participated in aducanumab studies. Similarly, ADUHELM ICARE AD-US is a phase 4 study planned to confirm clinical outcomes and generate data on long-term outcomes. It is expected to enroll about 6000 participants in the next 4 years, and it will likely be the main way in which patients will have access to it in the near future [84].

### Donanemab

Donanemab (N3pG) is a humanized IgG1 antibody that targets the pyroglutamyl E3 Aβ peptide (Aβ_p3-42_), a form of N-terminal truncated Aβ present only in stable Aβ plaques. Aβ_p3-42_ is a major toxic Aβ species prone to aggregation [85]. In the Aβ molecule, the aspartate and alanine at positions 1 and 2 are cleaved by an unknown mechanism. The glutamate at position 3 is then exposed and becomes vulnerable to pyroglutamylation by a glutamyl cyclase. Aβ_p3-42_ has high hydrophobicity, is prone to β-sheet stabilization with faster aggregation, is resistant to degradation, and becomes highly toxic. Donanemab was developed as a plaque-specific antibody, to overcome the problem that soluble Aβ forms could block recognition of deposited forms [86]. In an APP transgenic mouse model, a regular N-terminal antibody that binds both soluble and plaque Aβ, such as a bapinezumab murine analog, reduced brain Aβ burden when given early in the lifespan (e.g., 9-month-old) to prevent plaque buildup, but it had little effect at older ages (e.g., 18-month-old) once the plaque was formed [87]. In contrast, even extremely aged APP transgenic mice (> 23-month-old) experienced a reduction in Aβ plaques, upon treatment with donanemab. Plaque removal by donanemab is mediated by microglial activation [87].

Safety, tolerability, and pharmacokinetics of donanemab were tested in a phase 1a double-blind, randomized, placebo-controlled, parallel-group, single dose followed by a multiple dose study in MCI or mild dementia due to AD as confirmed with brain amyloid PET (*n* = 47, mean age 74, 2013–2016) [88]. Seven study arms tested intravenous donanemab in doses ranging from 0.1 to 10 mg/kg monthly for four doses. With this regimen, the half-life of donanemab was determined to be about 10 days. In the highest dose group, the amyloid PET (florbetapir) SUVR decreased from 1.65 to 0.26 at 7 months, which represented a 40% reduction in the Aβ burden. None of the participants developed ARIA, but donanemab was highly immunogenic, inducing anti-donanemab antibodies in 90% of participants [88]. In a subsequent phase 1 study (*n* = 63, 2015–2019) that recruited patients with the same characteristics, donanemab 10 or 20 mg/kg monthly for 16 months reduced amyloid by 90–100 centiloids, with several patients in the 20 mg/kg dose converting to negative brain amyloid status. About 25% of participants developed ARIA, mostly asymptomatic, and all developed anti-donanemab antibodies.

TRAILBLAZER-ALZ (2017–2021) was a multicenter, randomized, double-blind, placebo-controlled phase 2 trial in participants with MCI or mild dementia due to AD supported by a positive brain amyloid PET (*n* = 257, mean age 75, MMSE 20–28). For the first time, an AD clinical trial used flortaucipir PET to stage disease severity based on tau brain load. In addition to participants being required to have a positive brain amyloid PET (SUVR ≥ 1.17 or 36 centiloids), they were also required to have intermediate levels of brain tau (SUVR 1.10–1.46), compatible with Braak stages III and IV. The rationale for this was to exclude patients with low tau (Braak stages I and II), who were unlikely to progress during the trial, as well as those with high tau load (Braak stages V and VI), deemed to be too advanced to benefit from donanemab. Donanemab was given as an intravenous infusion of 700 mg monthly for the first 3 doses and then 1400 mg monthly for up to 72 weeks. The study design included blinded re-allocation of individuals receiving donanemab who had decreases in their brain amyloid PET burden at 24 or 52 weeks. Participants who had deeply negative amyloid PET (< 11 centiloids) or subthreshold amyloid levels (11–25 centiloids) in two consecutive scans were blindly switched to placebo. Those with subthreshold amyloid levels (11–25 centiloids) in the first scan were blindly switched to 700 mg monthly.

The primary outcome in TRAILBLAIZER-ALZ was the change from baseline in the integrated Alzheimer’s disease rating scale (iADRS) score at 76 weeks. The iADRS is an innovative metric that linearly combines ADAS-Cog13 and ADCS-iADL scores for a measure of both cognition and instrumental function, where scores range from 0 to 144, with lower scores reflecting worse disease. The primary outcome was met with a decline of − 10.06 points in the placebo group but only of − 6.86 points in the donanemab group. This is a 3.2-point (95% CI 0.1–6.2, *p* = 0.04) or 31.8% between-group difference. Group differences were not significant for secondary clinical outcomes (i.e., CDRsb, ADAS-Cog13, ADCS-iADL, and MMSE), but all showed trends for donanemab slowing the cognitive decline. The proportion of patients who completed the trial were similar in both groups, which makes unlikely that survivor bias explains these treatment responses [89]. The reduction in Aβ level as assessed by florbetapir PET was 85 centiloids greater in the donanemab group than in the placebo group. The percentage of participants in the donanemab group who had amyloid-negative status (defined as an amyloid plaque level of < 24 centiloids) at 76 weeks was 67.8%. Also, 27.4% and 54.7% of participants in the donanemab group had lowering Aβ enough to switch to placebo at 28 and 56 weeks, respectively. Participants with complete amyloid clearance showed significant decreases in parietal and frontal tau detected by brain tau PET, compared to placebo or participants with partial amyloid clearance groups. Donanemab was also associated with a 24% reduction in plasma P-tau217, whereas plasma P-tau217 increased by 6% in the placebo group. Donanemab was associated with ARIA. ARIA was seen in 40% of participants receiving donanemab, with about half of them being symptomatic (26.1%). In contrast, symptomatic ARIA-E was seen only in 0.8% of participants receiving placebo [90, 91]. Based on these data, the FDA granted donanemab a breakthrough therapy designation, which means an increased support for a more efficient approval and potential approval based on surrogate marker endpoints.

TRAILBLAZER-ALZ 2 (*n* = 1800, 2020–2025, NCT04437511) tested donanemab in a 76-week, randomized, placebo-controlled, double-blind, phase 3 study in MCI or mild dementia due to AD, confirmed by brain Aβ PET and by brain tau PET showing intermediate or high tau levels [92]. Based on a recent press release, the primary endpoint (iADRS) showed 35% slowing of decline with donanemab, compared to placebo. CDRsb, a secondary end point, showed 36% slowing of decline over 18 months. Participants on donanemab experienced a 39% lower risk of progressing to the next stage of disease, measured with the CDR global score, compared to placebo, and 40% less decline in ability to perform activities of daily living at 18 months. Thirty-four percent of participants in the intermediate tau load population achieved amyloid clearance at 6 months and 71% achieved clearance at 12 months based on the amyloid PET study. The incidence of symptomatic ARIA-E was 6.1% in the treatment group, whereas ARIA-H occurred in 31.4% in the donanemab group. Further testing of the clinical efficacy of donanemab is ongoing [93]. TRAILBLAZER-ALZ 3 (*n* = 3300, 2021–2027, NCT05026866) is a phase 3 study to evaluate the efficacy of donanemab in preclinical (i.e., intact cognitive function) AD. The primary outcome is time to clinical progression as determined by the CDR global score at 3.5 years. For the first time, high plasma P-tau217, reflective of brain amyloid and tau pathology burden, is being used as an inclusion criterion. TRAILBLAZER-ALZ 4 (*n* = 200, 2021–2024, NCT05108922) recently provided the first active comparator data on Aβ plaque clearance in patients treated with anti-amyloid immunotherapies. TRAILBLAZER-ALZ 4 is a phase 3, open label, superiority, head-to-head comparison of donanemab and aducanumab in participants with MCI or mild dementia due to AD, confirmed by brain amyloid PET. A primary outcome was the percent of participants who reach complete plaque clearance by brain Aβ PET in the overall or intermediate Aβ burden subpopulation at 6 months. All secondary outcomes were neuroimaging-related, and none were clinical outcomes. Brain Aβ clearance (< 24.1 centiloids) was achieved in 37.9% (25 of 66) of donanemab-treated participants compared to 1.6% (1 of 64) in the aducanumab group. In relation to baseline, donanemab reduced brain Aβ levels by 65.2%, compared to 17% with aducanumab. Donanemab, but not aducanumab, treatment significantly reduced plasma P-tau217 at 6 months compared to baseline. The safety profile of both treatments was consistent with their previously published studies. The incidence of ARIA was 25.4% (2.8% symptomatic, all ARIA-E) with donanemab and 26.1% (4.3% symptomatic, all ARIA-E) with aducanumab [94]. This incidence of ARIA is remarkably lower than the one observed in TRAILBLAZER-ALZ, which could be related to the time of adverse event measurements: 76 weeks in TRAILBLAZER-ALZ and 6 months in TRAILBLAZER-ALZ 4. Donanemab continues to be tested in TRAILBLAZER-ALZ 5, a registration trial for early symptomatic AD, currently enrolling in China, and TRAILBLAZER-ALZ 6, which is focused on expanding the understanding of ARIA through novel MRI sequences, blood-based biomarkers and different dosing regimens [93].

### Lecanemab

Lecanemab (BAN2401) is a humanized antibody that binds to soluble Aβ aggregates (oligomers and protofibrils) with high selectivity over monomer (> 1000-fold) and insoluble fibrils (approximately 10–15 fold) [95]. Lecanemab was developed after the observation that patients from a Swedish family with the Artic E693G *APP* mutation develop a clinical and neuropathological AD picture, with a high rate of soluble protofibril formation [96, 97]. In the ArcSwe transgenic mouse model, which carries both the Swedish and Artic *APP* mutations, treatment with mAb158, a murine version of lecanemab, decreased CSF Aβ protofibrils and prevented plaque formation in young mice, with a mechanism that involved astrocyte phagocytosis and prevention of Ab-induced neuronal death [95, 98, 99].

A phase 1 study (NCT01230853, 2010–2013) evaluated the safety and tolerability of lecanemab, with a multicenter double-blind randomized placebo-controlled design, in patients with mild-to-moderate dementia (Mean MMSE score 23, mean age 72, *n* = 80) due to AD. Lecanemab was given in parallel single and multiple ascending doses, from 0.1 mg/kg as a single dose to 10 mg/kg biweekly for 4 months. Each cohort consisted of eight subjects with two being randomized to placebo. The incidence of ARIA was low. Asymptomatic ARIA hemorrhage (ARIA-H) occurred in 3/60 (5%) of participants with lecanemab and 2/20 (10%) with placebo [100, 101].

Lecanemab was subsequently tested in a unique phase 2b (NCT01767311, 2012–2017), 18-month, multicenter, double-blind, placebo-controlled study in MCI and mild dementia due to AD, confirmed with a positive brain Aβ PET (*n* = 854, mean MMSE score 25.6, mean age 72). The study had an innovative design to increase efficiency that involved Bayesian response adaptive randomization across placebo and five lecanemab arms (2.5 mg/kg biweekly, 5 mg/kg monthly, 5 mg/kg biweekly, 10 mg/kg monthly, 10 mg/kg biweekly). The goal of the study was to identify the ED90 target dose (i.e., the simple treatment dose that achieves at least 90% of the modeled maximum treatment effect at 12 months) with a probability of at least 80%. Participants were randomized 3:1 to one of the five lecanemab regimens or placebo. Once a participant was allocated to a particular regimen, they stayed on that group for the duration of the study. However, allocation of subsequent participants to different groups was adjusted in response to frequent interim analyses occurring after the first 196 participants and then every 50 participants to evaluate the regimen showing the highest probability of being the maximal effective dose (i.e., better than placebo in preventing a change in the primary clinical endpoint by 25%). The primary clinical endpoint was the change in the AD composite score (ADCOMS) at 18 months. ADCOMS is a composite clinical score that measures cognition and instrumental function by incorporating elements of ADAS-Cog, MMSE, and CDR.

During the conduction of the study, after 375 patients had been randomized, regulatory input determined that the study could not enroll *APOE* ε4 carriers (approximately 70% of the overall study population) into the highest lecanemab dose (10 mg/kg biweekly), due to concern for increased risk of ARIA. In addition, the treatment with 10 mg/kg biweekly dose was immediately discontinued for all *APOE* ε4 carriers who had not reached 6 months of treatment. These actions caused a marked imbalance in the number of *APOE* ε4 carriers on 10 mg/kg biweekly and likely impacted the results of the study. The majority of participants were allocated to the second highest dose (10 mg/kg monthly). The 10 mg/kg biweekly (ED90) dose had only a 64% probability of being better than placebo with 25% less decline on ADCOMS at 12 months, instead of the pre-specified 80% probability. The probability of lecanemab 10 mg/kg monthly being the maximal effective dose was only 31.8%. The primary outcome was not met, but the study trends indicated potential for dose–response clinical efficacy. In addition, lecanemab reduced brain Aβ in a dose–response manner, with the highest decrease by − 0.31 PET SUVR at 18 months with lecanemab 10 mg/kg biweekly. This included several participants converting to brain amyloid PET negative by visual read. Moreover, 10 mg/kg biweekly lecanemab across the 18-month treatment period resulted in a reduction of clinical decline compared to placebo as demonstrated by ADCOMS (27%, with 97.7% probability to be superior to placebo), CDRsb (33%, with 96.4% probability to be superior to placebo), and ADASCog14 (56%, with 98.8% probability to be superior to placebo). The incidence of ARIA-E with lecanemab 10 mg/kg biweekly was 9.9% and with placebo 0.8%. For participants receiving lecanemab, the incidence of both ARIA-E and ARIA-H was higher in *APOE* ε4 carriers than in noncarriers [101].

Clarity AD (NCT03887455) was a multicenter, placebo-controlled, double-blind, parallel-group, randomized phase 3 study that tested lecanemab 10 mg/kg biweekly vs. placebo (1:1) in participants with MCI or mild dementia due to AD supported by a positive brain Aβ PET (*n* = 1795, mean CDRsb 3.2 points, 2019–2025). The primary outcome was the change in CDRsb at 18 months. The CDRsb change was 1.21 points with lecanemab and 1.66 with placebo. This represents a between-group difference of − 0.45 CDRs points (95% CI − 0.67 to − 0.23, *p* < 0.001) or 27%. Lecanemab also induced greater reductions in Aβ burden compared to placebo (difference − 59.1 centiloids, 95% CI − 62.2 to − 55.6, *p* < 0.01), as well as ADAS-cog14 (difference − 1.44, 95% CI − 2.27 to − 0.61, *p* < 0.001), ADCOMS (difference − 0.05, 95% CI − 0.074 to − 0.027, *p* < 0.001), and ADCS MCI-ADL (difference 2, 95% CI 1.2 to 2.8, *p* < 0.001). The incidence of ARIA-E was 12.5% with lecanemab and 1.7% with placebo. The incidence of ARIA-H was 17% with lecanemab and 8.7% with placebo. The incidence of isolated symptomatic ARIA-H was 0.7% in the lecanemab group and 0.2% in the placebo group, while the incidence of symptomatic ARIA-E was 2.8% in the lecanemab group and 0 in the placebo group [102]. Based on these data, lecanemab (Leqembi™, Eisai) was approved on January 6, 2023, by the FDA for the treatment of MCI or mild dementia due to AD. Similar to aducanumab, lecanemab was approved under the accelerated approval pathway for conditions where there is an unmet medical need and a candidate drug shows an effect on a surrogate endpoint that reasonably predicts a clinical benefit.

AHEAD 3–4/5 (NCT04468659) is a multicenter, randomized, double-blind, placebo-controlled phase 3 study of lecanemab in cognitively healthy individuals with either intermediate (A3) or high (A4-5) brain amyloidosis determined by brain Aβ PET that is currently open to enrollment (*n* = 1400, 2020–2027). A3 (*n* = 400) enrolls individuals with brain Aβ at subthreshold levels (20–40 centiloids). Participants are randomized 1:1 to placebo or lecanemab 10 mg/kg monthly for 4 years, and the primary endpoint is a reduction in brain Aβ. A4/5 (*n* = 1000) enrolls participants with high Aβ (> 40 centiloids). They are randomized 1:1 to placebo or lecanemab 10 mg/kg biweekly for 2 years, followed by lecanemab 10 mg/kg monthly until 4 years. The primary endpoint is the change in cognitive function, as measured by an optimized PACC that includes measures of semantic processing (PACC-5), which may have improve sensitivity to cognitive change in preclinical AD [103]. Current additional studies with lecanemab include a 5-year phase 2 long-term extension and a 4-year phase 3 long-term extension in early AD, and a 4-year dominantly inherited Alzheimer network trials unit (DIAN-TU) next generation trial in autosomal-dominant genetic AD.

### Emerging Anti-Amyloid Immunotherapies

A number of additional anti-amyloid immunotherapies are currently in the therapeutic development pipeline. *ACU193* is a monoclonal antibody that selectively binds soluble Aβ oligomers. It is currently being tested in a multicenter phase 1 trial in MCI and mild AD [104]. *Trontinemab* is “brain shuttle” gantenerumab, with improved blood–brain barrier penetration, based on interaction with the transferrin receptor on endothelial cells. The improved bioavailability was tested in a phase 1 study in healthy participants and is currently being tested in another phase 1 study in PET-amyloid positive MCI and mild AD [105]. *Remternetug* is an enhanced analogous of donanemab that also targets N-terminal pyroglutamated Aβ. It is currently being tested in a phase 1 study in MCI or mild AD, with preliminary data showing that a dose of 2800 mg IV monthly converts amyloid PET positive participants to amyloid PET negative in 3 months. Remterneteug is also being tested against placebo in TRAILRUNNER-ALZ1, a phase 3 study in early symptomatic AD, with intravenous or subcutaneous administration for 1 year [106].

## Opportunities for Future Clinical Trials of Anti-Amyloid Immunotherapies

### Improving Diversity, Equity, and Inclusion

Diverse societies have a strategic position and a responsibility to explore socioeconomic factors that influence the impact of emerging therapies in AD. Unfortunately, therapeutic development in AD has had limitations related to participation of diverse populations in clinical trials. Trials of anti-amyloid therapies have lacked participation of non-White individuals to reflect the demographics of communities in which clinical trials are conducted. For example, EMERGE and ENGAGE, the phase 3 trials that tested aducanumab, were conducted globally and enrolled a total of 3285 participants, but only 19 (0.5%) of them were Black, and only 104 (3.1%) were Hispanic. These concerning limitations have modestly improved in more recent trials with donanemab and lecanemab, but enrollment of historically minoritized populations has not significantly improved. This lack of diversity seems to be pervasive worldwide, as illustrated by low enrollment of Black, Asian, and Hispanic participants in international multicenter trials, including EMERGE, ENGAGE, and TRAILBLAZER-ALZ [79, 90]. Identifying barriers for a more diverse participation and innovation in recruitment science are crucial areas of opportunity to generate clinical trials data that are generalizable and more likely to have a distributed benefit from drug development. Barriers for diverse participation include micro- (e.g., participant education, individual attitudes about research, individual views about cognitive health and dementia), meso- (e.g., cultural and language competency of research staff, design of trials that do not account the social needs of diverse populations, physical access to research centers), and macro- (e.g., lack of incentives for clinical trialists to recruit diverse populations, insufficient funding opportunities for diversity research)level factors [107]. Systemic racism remains an important macro-level barrier, and clinical trialists in AD should remain aware of potential consequential bias emerging from it. Although race is a social construct, tackling barriers linking race to social determinants of health has the potential of having a significant impact on deployment of anti-amyloid immunotherapies. For example, amyloid status may be linked to social determinants of health and co-segregate with race. In A4 study, reduced brain amyloid has been detected in self-identified non-Hispanic Black participants. However, a greater proportion of non-Hispanic Black participants did not pass the initial criteria for the A4 clinical trials enrollment, compared to White participants [108, 109]. Trials of anti-amyloid therapies could adopt explicit antiracist strategies such as (1) establishing relationships with the community to provide information and build trust, (2) adapting infrastructure and practices to address systemic inequities, (3) setting aggressive enrollment goals for diverse populations, (4) avoiding restrictive eligibility criteria, (5) introducing requirements to disclose detailed racial/ethnic composition of study samples, (6) modifying third-payer policies that restrict therapy coverage for patients not able to enroll in observational studies or registry, commonly run by institutions with weak track records for enrollment diversity, and (7) improving representation of historically minoritized groups in the executive workforce responsible for making funding decisions and regulatory policies [107].

Some examples of progress on this front are noticed in ongoing studies. For example, the aducanumab post-marketing surveillance study ADUHELM ICARE is expected to enroll 6000 participants in the next 4 years, with a goal of at least 16% Hispanics and Black/African American patients. This goal is about a twofold increase in recruitment of Hispanics and about a fivefold increase for Black/African Americans, compared to recently completed phase 2 and 3 anti-amyloid immunotherapy trials. Similarly, in clarity AD, approximately 25% of the total US enrollment included Hispanic and African American participants. TRAILBLAZER-ALZ 3 is overcoming physical and schedule barriers through incorporation of remote clinical assessments supported by telehealth and app-based technology. To a similar effect, AHEAD 3–4/5 is partnering with commercial transportation vendors to facilitate physical access by offering participants a door-to-door service from home to the research center and back. Innovation in recruitment science to address meso- and macro-level barriers for recruitment is also being promoted by AHEAD 3–4/5. This trial has a diversity, equity, and inclusion arm with dedicated intellectual effort and funding to increase recruitment of historically minoritized groups. In addition, this trial is serving as a context for the multidisciplinary study of the effects of leveraging the national infrastructure and goodwill of trusted organizations, such as the Alzheimer’s Association and the National Association of Hispanic Nurses, to increase recruitment of Hispanics. Facilitating the administration of investigational products at home can also result in convenient scenarios to overcome access barriers to which disadvantaged groups are particularly sensitive. This includes, exploring the feasibility of home infusions or the introduction of anti-amyloid immunotherapies that can be administrated via subcutaneous injection at home. This will require careful consideration of potential barriers to home administration of anti-amyloid therapies, such as the risk of hypersensitivity reactions occurring in a non-acute setting or the need for funding sources to pay for the infrastructure to provide home infusions. Relaxation of inclusion criteria could open the doors of clinical trials to a wider population. As another advancement in helping the population to have better access to emerging therapies, the Veterans’ health administration (VA) has recently announced that it will provide coverage for lecanemab [110]. Potential participants in AD trials have been historically excluded if cognitive or neuropsychological testing is not possible because the participant is not able to read or write or if they do not meet memory tests inclusion criteria scores, because they present with non-amnestic forms of AD. The development of better tools to screen and measure cognitive and functional outcomes could open the door to participants regardless of literacy or AD phenotype. However, since AD can be a heterogeneous disease, and therefore, therapeutics may be more beneficial for some forms of AD than others, including many AD phenotypes may reduce the power of studies to detect benefit in the phenotype most amenable to that treatment. As such, including participants with different AD phenotypes would also necessitate increasing overall enrollment [111]. Related to this, two additional strategies with potential to improve equity and inclusion are the use of decentralized tools for recruitment and innovation in clinical trial design. Improved access to recruitment could be achieved through the use of tools like the GeneMatch or TRC-PAD databases, which are accessible by volunteers potentially at a global scale and can create customizable trial-ready cohorts of participants [112]. Enrollment of disadvantaged groups may be improved by wider adoption of screening tools for amyloidosis that rely on easily deployable tests in the community (e.g., plasma P-tau217 or plasma Aβ_40_/Aβ_42_) [113]. Innovation in clinical trial design may shorten the time and the size of cohorts needed to determine if therapies are safe or effective and accelerate their approval and availability to the general population. Innovative study designs have shown success in cancer therapy research, including trials with Bayesian response adaptive randomization, basket trials (i.e., testing one drug or placebo in different phenotypes or diseases), and platform trials (i.e., several drugs are tested head-to-head in the same population with a favorable active treatment group allocation by the use of a pooled placebo group [114] (Tables 3 and 4).

**Table 3:** Incidence of ARIA with 3rd generation anti-amyloid immunotherapies. Data from EMERGE, TRAILBLAZER-ALZ, and Clarity AD trials

	***Aducanumab***		***Donanemab***		***Lecanemab***	
	Most effective dose	Placebo	Most effective dose	Placebo	Most effective dose	Placebo
All ARIA	41.3%	10.3%	38.9%	8%	26.6%	9.4%
ARIA-E	35.2%	2.7%	26.7%	0.8%	12.6%	1.7%
ARIA-H	19.1%	6.6%	30.5%	7.2%	14.0%	7.7%
Discontinuation	6.2%	0.6%	15%	4.8%	6.9%	2.9%
Death	1%	0.9%	0.8%	1.6%	0.7%	0.8%

**Table 4:** Innovations facilitated by anti-amyloid immunotherapies

*Current*
Use of *APOE* genotype to stratify titration regimens
Use of amyloid PET scan to confirm AD pathology and to monitor the drug efficacy
Use of plasma P-tau217 and plasma Ab42/Ab40 to screen participants prior to enrollment and to monitor their response to treatment
Use of flortaucipir PET to stage disease severity based on Braak staging as a screen test prior to enrollment
Introduction of novel clinical scales with more sensitivity for clinical change, especially in early stages of the disease
Targeting population at an earlier stage of the disease course such as cognitively healthy individuals
Use of innovative trial designs (e.g., active randomization, Bayesian response adaptive randomization, switch to placebo arm upon evidence of amyloid lowering on PET scan)
Stop rules and decision-making protocols to manage ARIA
Introduction of measures to improve inclusion and diversity (e.g., use of trial-ready registries, coverage of transportation to research center)
Testing of alternate routes for drug delivery (i.e., subcutaneous)
Disclosure of brain amyloid status to cognitively healthy individuals
*Future*
Novel clinical trial designs (e.g., platform trials, basket trials)
Remote clinical outcome assessment supported by telemedicine and mobile apps
Biomarker-based identification of responders vs. non-responders (e.g., fluid biomarker, neurophysiological)
Further efforts to improve inclusion and diversity (e.g., presence of academic and industry teams in the communities)
Deployment of home infusions
Testing of anti-amyloid immunotherapies plus drugs with other mechanisms of action (e.g., anti-tau immunotherapies, anti-sense oligonucleotides or small molecules)

### Emerging Ethical Considerations in Trials of Anti-Amyloid Immunotherapies

Upcoming trials of anti-amyloid immunotherapies in the next few years will make the field face ethical dilemmas around the disclosure of amyloid status to clinical trials participants. Disclosure of a positive brain amyloid status, as revealed by CSF studies or brain amyloid PET is already perceived as a potential source of psychological distress to patients with MCI or dementia. Disclosure of positive brain amyloid status to *cognitively asymptomatic individuals* is currently not done in clinical practice, since AD biomarkers are not indicated in this population. In addition, there has been concern that disclosure of positive brain amyloid status could have catastrophic psychological consequences for cognitively healthy people (e.g., “If I knew I will get Alzheimer’s, I would kill myself”). As the field of AD research is moving to the detection of ongoing AD pathophysiology in the earliest possible stages, scenarios where disclosure will be considered are expected to be common, especially with the availability of anti-amyloid therapies to be used in pre-symptomatic AD stages [115]. Landmark studies such as A4 or AHEAD A3-4/5 are exploring the safety and feasibility of disclosure of amyloid status to cognitively healthy individuals, by systematically studying the psychological consequences of disclosing this information, as well as standardization of ways to assess and mitigate risk, deliver information, and conduct proper follow up. AHEAD A3-4/5 is exploring a structured approach to disclosing amyloid status that comprises education and informed consent, psychological well-being and suicidality assessment, and post-disclosure follow up [116]. Pre-disclosure education emphasizes a teach back method (e.g., “In your own words, what does it mean to have a positive amyloid PET result?”) and appreciation of information (e.g., “tell me what you know about an amyloid PET,” “Why are you interested in having an amyloid PET? If your amyloid PET is abnormal, what would that mean to you?”). Assessment of psychological well-being emphasizes the use of structured tools for anxiety, distress upon traumatic events, and suicidality. Follow up highlights the need for a protocol to increase monitoring of psychological status if affective changes or suicide risk is detected [115].

Reassuring A4 data show that disclosure of brain amyloid PET results to cognitively healthy individuals does not result in increased suicidality, depressive symptoms or anxiety, as measured by structured clinical tools [116]. A4 data also revealed that when a participant learns about their positive amyloid status, they gain certainty that someday they will get AD, a concern that is prioritized among other health concerns, compared to individuals who learned about a negative amyloid status [116]. Nevertheless, individuals who learn about a positive amyloid status are also more likely to engage in healthy lifestyle changes, such as improved exercise, diet, cognitive activity, mediation, socializing, and other quality of life activities [117, 118]. The relevance of this is appreciated in the context of longitudinal observational studies of cognitively healthy people with a positive amyloid status, which have shown that higher physical activity is associated with a stable performance in cognitive testing and brain volume, compared to cognitive decline and brain atrophy in default-mode brain regions, vulnerable in AD, in brain amyloid positive individuals with low physical activity [119].

With these reassuring data, we expect that disclosure of amyloid status to cognitively healthy individuals will enter primary care and specialty practice within the next decade. New tools such as blood tests for AD may make it easier for primary providers to screen for the disease, and there will be a need for non-specialty providers to gain knowledge about interpretation and disclosure of results. Discussions around amyloid status will become ethically compelling, when considering that not only emerging anti-amyloid immunotherapies therapies but also lifestyle interventions may impact the trajectory of the disease. Cognitively healthy individuals will be more interested in preventive care and will be more likely to establish care with healthcare providers for this purpose. Neurologists will likely face new challenges not only as diagnosticians or care providers but also as preventionists. Moreover, healthcare systems and third payers will have to address new care challenges related to cognitively healthy people. The emergence of anti-amyloid therapies with the ability to change the trajectory of the disease will likely reshape clinical practice not only to include disclosure of amyloid status, the use of anti-amyloid therapies, and management of complications, and yet-to-discover drug interactions and its clinical challenges but also to implement a closer cognitive surveillance in older adults and lifestyle changes for a disease that otherwise causes significant burden on individuals and their families.

## Conclusion

Anti-amyloid therapies are the mainstream line of clinical research in AD therapeutics. Phase 3 clinical trials of anti-amyloid therapies have been the vehicle for significant innovation in AD clinical science and study designs, and even more progress is expected with ongoing and future studies. Encouraging progress, including recent FDA approval of aducanumab and lecanemab, two disease-modifying therapies against AD, is the product of a better understanding of the Aβ-mediated AD pathophysiology, with an evolving concept of AD as a pathobiological entity, and the emergence of better clinical tools, including more specific anti-Aβ antibodies and the use of PET for detection of brain Aβ in vivo. Development of better anti-Aβ immunotherapies will require sustained innovation in clinical trial design, with consideration of efficiency and equity, and addressing emerging clinical, ethical, and logistical challenges. In 2023, the horizon of AD clinical care is promising and will likely be an inflection point in the practice of neurology.

## Data Availability

No original data are presented. All data are referenced.
